# The Clinical Significance and Transcription Regulation of a DNA Damage Repair Gene, SMC4, in Low-Grade Glioma *via* Integrated Bioinformatic Analysis

**DOI:** 10.3389/fonc.2021.761693

**Published:** 2021-11-11

**Authors:** Yan Wang, Zhisheng Wu

**Affiliations:** Department of Neurology, First Hospital of Quanzhou, Quanzhou, China

**Keywords:** DNA damage repair, SMC4, bioinformatic, low-grade glioma, transcriptional modulation

## Abstract

Glioma is the most common type of malignant tumor in the central nervous system with an unfavorable prognosis and limited treatment. In this study, we are devoted to addressing the prognostic value of DNA damage repair-related genes in low-grade glioma (LGG). We plotted the landscape of DNA damage repair (DDR)-related genes and identified SMC4 as an independent prognostic marker with integrated bioinformatics analysis, which is overexpressed in different histologic subtypes of glioma. We observed that SMC4 expression is elevated in recurrent LGG patients or those with advanced histologic staging. SMC4 depletion inhibits proliferation and induces increased replication damage in LGG cells. Lastly, we predicted and validated the transcription modulation of SMC4 by a transcription factor, MYB, at the -976bp~ -837bp of the SMC4 promoter region in LGG cells. Together, our study identified SMC4 as a potential prognostic biomarker for LGG patients, which functions to promote cell proliferation by repairing replication damage and the expression of SMC4 could be transcriptionally regulated by MYB.

## Introduction

Glioma is the most prevalent primary malignant brain tumor in adults and is canonically categorized into 4 different stages, among which grade II and III frequently possess IDH mutation, collectively termed as low-grade glioma (LGG) (1). And LGG constitutes a heterogeneous group of neuroepithelial neoplasms, including astrocytoma, oligodendroglioma, and oligo-astrocytoma (2, 3), etc. While the proliferation rate is slower, the prognosis of LGG is poor due to its infiltrative nature and aggressive behavior (4). While multiple molecular subtypes and clusters of glioma have been suggested within decades, the effective treatment for LGG remains limited (5, 6). In this study, we sought to find potential targets for the LGG treatment.

Multiple biological processes in cells produce reactive oxygen species (ROS) oxidized DNA lesions, most commonly observed in mitochondria, and cause numerous oxidized DNA lesions. And the accumulation of DNA lesions would potentially hamper the physiological function and survival of the cell (7). To solve this, many cells from diverse histological origins are conserved in the DNA damage repair (DDR) pathways (7, 8). As one of the most fundamental functions of cells, DDR pathways are mainly comprised of 5 pathways, including mismatch repair (MMR), base excision repair (BER), homologous recombination (HR), non-homologous end joining (NHEJ), and nucleotide excision repair (NER). And most pathways are consistently activated in highly proliferative cells and metabolic active cells, especially in cancer (9–11). The role of DDR, especially mismatch repair pathways in several cancer types has been discussed, the function of these genes in LGG has not been unveiled (12–14). Also, while targeted therapies inhibiting the key enzymes in the DDR pathways have been raised for the treatment of cancer, such as MTH1 inhibitor, PARP inhibitor (15, 16), etc., validated targets for LGG patients are still limited and the search for druggable key enzymes with potential prognostic values are urgent (15, 16). While the gene expression and cellular landscape of several brain tumors have been unveiled with single cell sequencing, the DNA damage repair landscape of glioma is still lacking due to the relatively low expression level.

Structural maintenance of chromosome 4 (SMC4) is a critical member of the SMC family and is considered to facilitate the sister-chromatid condensation and mitosis (17). SMC4 is highly conserved across species and is observed to be overexpressed in multiple solid tumors, including hepatocellular carcinoma, colorectal cancer, and prostate cancer, etc. (18–20). Recent studies have unveiled its correlation with tumor proliferation, differentiation, and vascular invasion in different cancers and suggested that miR-219 might be responsible for the regulation of SMC4 expression (18, 21). However, the expression of SMC4 in brain tumors, such as LGG, and its canonical regulation by transcription factors or DNA methylation status are unknown.

## Material and Method

### Data Collection

mRNA sequencing data, DNA methylation data, and clinical data of low-grade glioma patients and other cancer types were acquired from the TCGA LGG database (https://tcga-data.nci.nih.gov/), GEO database (GSE147352, GSE78895, and GSE152071, etc., https://www.ncbi.nlm.nih.gov), Oncomine database (https://www.oncomine.org/resource/main.html) and GEPIA database (http://gepia.cancer-pku.cn/index.html). Expression patterns of normal brain tissues were obtained from TCGA database (https://xenabrowser.net/datapages/).

Stemness score was acquired from the TCGA PAN-CANCER database (https://xenabrowser.net/datapages/). Telomere length for low-grade glioma patients was acquired from previous research (22). IDH mutation and 1p19q co-deletion data were obtained from China Glioma Genome Atlas (CGGA, http://www.cgga.org.cn/) Cell line sequencing data and IC50 for TMZ were obtained from Genomics of Drug Sensitivity in Cancer (http://www.cancerrxgene.org/).

### Pathway Enrichment Analysis

Kyoto Encyclopedia of Genes and Genomes (KEGG) and Gene Ontology (GO) enrichment analysis are traditional bioinformatics tools to map a certain group of genes to different pathways and to calculate corresponding odds ratio and significance. GO enrichment analysis is composed of three diverse parts: biological process (GO_BP), cell component (GO_CC), and molecular function (GO_MF). Both KEGG and GO analysis was performed with the Enrichr website (http://amp.pharm.mssm.edu/Enrichr/).

GSEA was performed with the GSEA 4.0.3 software (http://www.gsea-msigdb.org/gsea/index.jsp). GSEA analysis was performed to understand the activation status of a certain pathway with a defined set of genes.3 major GO terms, including GO_BP, GO_MF and GO_CC, and KEGG pathways were included in the analysis. Pathways and genes showed statistical significance and concordant differences were considered altered pathways.

### Kaplan-Meier Analysis

Kapan-Meier analysis was performed in this study to compare the next (OS), disease-specific survival (DSS), and progression-free interval (PFI) in glioma patients.

Kaplan-Meier analysis was performed with Graphpad (https://www.graphpad.com/), SPSS (https://www.ibm.com/products/spss-statistics), or GEPIA to calculate Hazard ratio and log-rank significance in different groups of glioma patients. Besides, Kaplan-Meier analysis was performed with SPSS to stratify patients with respect to histological grade and new tumor events in low-grade glioma patients.

The cut-off value for all the Kaplan-Meier analyses in this article is defined by the median of its corresponding expression level. Gene expression higher than the cut-off value is defined as a high-expression group, vice versa.

### Multiple Variate Cox Regression

Multiple variate Cox regression was performed with SPSS software to select independent factors for the prognosis of glioma patients. The forward stepwise method was used in the multivariate Cox Regression model with p < 0.05 to enter and p < 0.10 to exit.

Independent factors which are significant in predicting OS or PFI were selected and the risk score for both OS and PFI were calculated for glioma patients.

### Heatmap and Hierarchical Clustering

Heatmap with hierarchical clustering was achieved with MeV software (https://sourceforge.net/projects/mev-tm4/). For Heatmap, the expression level of each gene was normalized to the median in each patient and the color scale was normalized to -2 ~ 2. Hierarchical clustering was performed with the Euclidean clustering method with complete linkage and optimized gene/sample order.

Heatmap and the following hierarchical clustering enabled us to directly visualize and comprehend the expression pattern of a certain cluster of genes or samples.

### Correlation Analysis and Student’s T-Test

Correlation between two groups of samples (including **Figures 2H**, **4D, G**, **6B**, **5F, G**, and **Supplementary Figures 3C–E**) was performed with SPSS and plotted with Graphpad. Linear regression was performed with Graphpad with 95% CI labeled.

Student’s t test (including **Figures 2F**, **3B, C, E, F**, **4E**, **5A, C, D, F**, **6A** and **Supplementary Figure 2E**) was performed with Graphpad.

### Prediction of the Transcriptional Modulation of SMC4

To further explore the potential transcriptional modulation of SMC4 in low-grade glioma patients, we first acquired the DNA sequence of the SMC4 promoter (-2000bp ~ -1bp) in the UCSC database (http://www.genome.ucsc.edu/index.html). Then we predicted the binding affinity of all the known human transcription factors listed in the PROMO database (http://alggen.lsi.upc.es/recerca/frame-recerca.html).

Next, the transcription factors with at least 2 binding site < 5% dissimilarly was selected as potential transcription factors for the modulation of SMC4. We analyzed the expression level of SMC4 and candidate transcription factors and transcription factors with Pearson R > 0.4 or < -0.4 were selected. And we validated our findings in the GEPIA database and 3 datasets in the GEO database.

### Cell Proliferation Assay and Dose-Response Analysis

Two glioma cell lines, LN229, and SW1088 were obtained from Dr. L. Yang from the Third Affiliated Hospital of Harbin Medical University. Two cells were cultured in DMEM supplemented with 10% FBS and 1% penicillin and streptomycin at 37 degrees in a humidified chamber with 5% CO2.

Cells were seeded in the 96-well plate at 2000 cells/well and cultured for 5 days. The proliferation curve was plotted with CCK8 (Cell Counting Kit-8).

Dose-response for temozolomide was performed as previously described (23). Briefly, SW1088 cells were transfected with siCtrl, siSMC4 #1 and siSMC4 #2 on day 0. Cells were re-suspended and seeded in a 96-well plate at 5000 cells/well on day 1 and treated with an increased concentration of temozolomide for 3 days. Cell number was quantified with CCK8 at the end of the experiment.

### Constructs and Transfections

Specific target shRNAs were subcloned into lentiviral vector pLKO.1 (Shanghai Genomeditech) and a non-target shRNA was used as a negative control. 2 shRNA sequences are: shSMC4 #1: 5’- GCATGTTAAGGAAACTCAGCC-3’ and shSMC4 #2: 5’- GCAGCGCCTCATCAAGAAAGT-3’ (24).

MYB-overexpression construct was purchased from Sigma-Aldrich, as previously described (25) and an empty vector was used as a negative control. Viral particles were packaged in 293T cells and used to infect glioma cell lines in the presence of 8 µg/ml polybrene, followed by puromycin selection.

### Antibody and Reagents

The following primary antibodies were used:

SMC4 ab17958 Rb Abcam WB
γH2AX 9718S Rb CST IF
MYB ab109127 Rb Abcam WB
Flag 14793S Rb CST ChIP

The following secondary antibodies were used:

Alexa Fluor^®^ 488 anti-Rabbit ab150077 Goat Abcam IF
HRP-labeled Anti-Rabbit A0208 Goat Beyotime WB

Temozolomide was purchased from Selleck (https://www.selleck.cn/).

### Immunofluorescence and EdU Staining

Immunofluorescence was performed as previously described (23). Briefly, cells were fixed with 3.7% PFA and permeabilization with 1% Triton-X100, then cells were washed with PBS twice and blocked for 1h in blocking solution. Cells were incubated with primary antibody (diluted in blocking solution) and the secondary antibody, followed by washing with PBS-Tween 20. Last, cells were counterstained with DAPI before image acquisition.

EdU staining kit was purchased from Beyotime (https://www.beyotime.com/). Cells were incubated with 1:1000 EdU for 30min and fixed with 3.7% PFA. Cells were then permeabilized, blocked, and incubated with primary/secondary antibody, after which EdU-Click reaction is performed according to the manufacturer’s protocol, as previously described (23). After 5 times washing with PBS, cells were counterstained with DAPI, and images were acquired. The colocalization results were analyzed with a Cell profiler.

### Chromatin Immunoprecipitation (ChIP)

ChIP was performed with a Chromatin Immunoprecipitation kit (Merck Millipore, MA, USA) according to the manufacturer’s instructions. The enrichment of the DNA template was analyzed by conventional quantitative PCR, using primers specific for each target gene promoter. Primers sequences were listed as follows:

Primer for the SMC4 promoter region -1024 ~ -1163,
F: 5’- TTCTCGGGGAACAGGAATCG-3’;
R: 5’- CGACTCGCCAGTGTTATGGT-3’;

### Quantitative Polymerase Chain Reaction (qPCR)

Qiagen RNA isolation kit and reverse transcriptase were used to extract total RNA from tissues and cultured cells and to synthesize complementary DNA.

qPCR was performed using SYBR Premix Ex Taq (Takara) in a Bio-Rad CFX-96 Real-Time PCR detector. Primers for MYB and SMC4 are as follows:

h_MYB_F: 5’-ATCTCCCGAATCGAACAGATGT-3’
h_MYB_R: 5’-TGCTTGGCAATAACAGACCAAC-3’
h_SMC4_F: 5’-TTGTCATGCACTGGACTACATTG-3’
h_SMC4_R: 5’-TTTTTCGCCCATACAGCCATC-3’
h_ GAPDH _F: 5’- GCCATGTAGACCCCTTGAAGAGC-3’;
h_ GAPDH _R: 5’- ACTGGTTGAGCACAGGGTACTTTAT-3’.

### Dual-Luciferase Reporter Assay

Dual-luciferase reporter assay was performed according to the manufacturer’s protocol (Beyotime, RG027). Briefly, cells were co-transfected with firefly luciferase control plasmid along with renilla reporter plasmid at a ratio of 10:1. After 48 hours, cells were harvested and lysed in a lysis buffer. The activity of luciferase was detected by the Dual-Luciferase Reporter Assay System. The results were normalized to the renilla activities.

Promoter region used were: GTTAAAAACCCACTAAAACTAAGTTCTCGGGGAACAGGAATCGCCAAACTTGCTTACTAATTCTAATTATGCCTTACCCAGGGCAATGTATACAAACACTACTTAGGGAATATTTGCTGATCGATAATTTCCTCATAAAAATAACCATAACACTGGCGAGTCGTTTTTTCCACTAAAGAGCCTTTTAGTCAAATTATTTT.

## Result

### The Landscape of DNA Damage Repair in Glioma Patients

**Figure 1:**
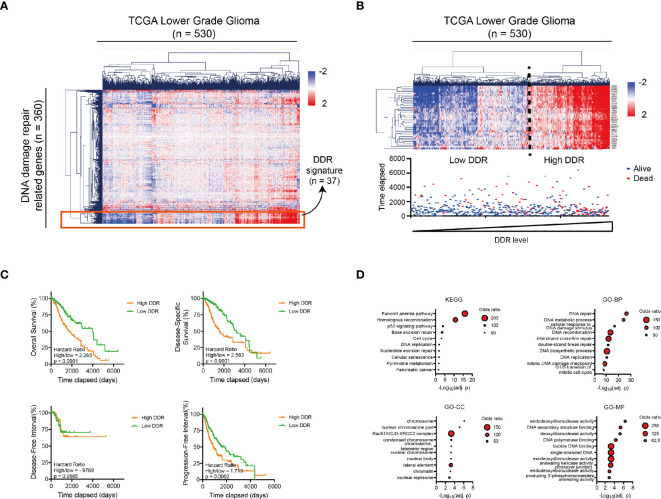
The DNA damage repair landscape of low-grade glioma patients in TCGA database. **(A)** Heatmap and Hierarchical Clustering showing the heterogenous expression of 360 DNA damage repair-related genes in 530 glioma patients in the TCGA database. A sub-cluster of highly homogeneously expressed genes is marked in the red square. **(B)** Top: heatmap and Hierarchical Clustering of the glioma patients with 37 DNA damage repair signature genes (n = 530) from **Figure 1A**. Patients can be divided into high or low DDR level group according to the Hierarchical Clustering result. Bottom: vital status of the glioma patients with different DNA damage repair levels as presented in the top figure (n = 530). Deceased patients are labeled in red and censored/living patients are labeled in blue. **(C)** Kaplan-Meier analysis showing the OS, DSS, DFI and PFI of glioma patients with high *vs.* low DNA damage repair level according to the median (n_high_ = 265, n_low_ = 265). **(D)** Bubble diagram showing the KEGG and GO analysis results (including GO_BP, GO_MF and GO_CC) for 37 DNA damage repair signature genes. The size of each point represents the number of genes in the representative pathway. DDR, DNA Damage Repair; OS, Overall Survival; DSS, Disease-Specific Survival; DFI, Disease-Free Interval; PFI, Progression-Free Interval; GO, Gene Ontology; BP, biological process; CC, cellular component; MF, molecular function; KEGG, Kyoto Encyclopedia of Genes and Genomes.

We started to illustrate the landscape of DNA damage repair (DDR) -related genes in glioma patients. A total of 360 genes involved in the DDR pathways were defined as previously reported (26). The mRNA sequencing data of DDR genes were collected from TCGA Low-grade Glioma (LGG) database. While many of the DDR-related genes are heterogeneously expressed in glioma patients, a cluster of genes (n = 37) are simultaneously expressed across glioma patients in the Euclidean hierarchical clustering, which is defined as DDR signature genes (**Figure 1A**).

LGG patients were subsequently clustered into high and low DDR groups according to the DDR signature (**Figure 1B**, the top panel) and we noticed that the vital status of glioma patients was altered in the high DDR group (**Figure 1B**, the bottom panel). Consistently, LGG patients with higher DDR signature gene expression showed significantly unfavorable prognosis in the overall survival (OS), disease-specific survival (DSS), disease-free interval (DFI), and progression-free interval (PFI) of glioma patients (**Figure 1C**).

To interpret the prognostic value of DDR signatures in LGG patients, we performed KEGG and Gene Ontology analysis with all the DDR signature genes. Results showed the enrichment of the Fanconi Anemia and the Homologous Recombination pathway in KEGG, the enrichment of the DNA repair, DNA metabolic process in GO_BP (biological process) term, the enrichment of Rad51B/C/D-XRCC2 complex in GO_CC (cell component) term and the enrichment of Endodeoxyribonuclease activity in GO_MF (molecular function) term (**Figure 1D**). These results indicated that the activation of DNA damage repair pathways, especially the Fanconi Anemia and the Homologous Recombination pathways, etc., was responsible for the unfavorable prognosis of the glioma patients.

### The Identification of SMC4 as a Prognostic Marker for Glioma

Kaplan-Meier analysis showed all the DDR signature genes, together with two clinical factors, neoplasm histologic grade and new tumor event after initial treatment could affect the OS and PFI of the glioma patients respectively (**Supplementary Figures 1A, B**).

**Figure 2:**
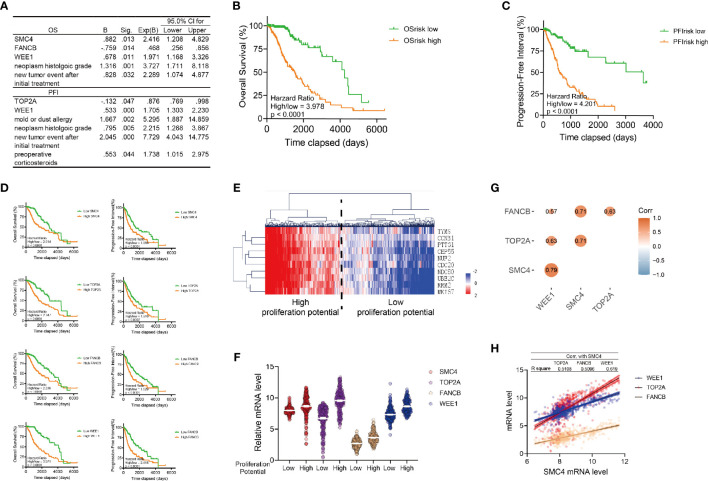
Identification of SMC4 as a prognostic marker of low-grade glioma patients. **(A)** Table showing the results of the multivariate Cox Regression for the OS (the top table) or PFI (the bottom table) of LGG patients in TCGA database with Forward Stepwise method in SPSS. Genes or clinical signatures identified as risk factors are listed in the table. The B factor, p value (Sig.), expected B factor (with 95% CI) are provided for each risk factor. **(B, C)** Kaplan-Meier analysis comparing the OS **(B)** and PFI **(C)** of high or low risk LGG patients. 530 patients were enrolled and divided into high/low-risk groups according to the median of OS risk score or PFI risk score (n_high_ = 265, n_low_ = 265). **(D)** Kaplan-Meier analysis comparing the OS and PFI in glioma patients according to the expression levels of SMC4, TOP2A, FANCB and WEE1. 530 patients were enrolled and divided into high/low-risk groups according to the median of their respective risk score (n_high_ = 265, n_low_ = 265). **(E)** Heatmap and Hierarchical Clustering showing the proliferation potency of glioma patients. Patients were divided into high or low proliferative potential group according to the Hierarchical Clustering results. Proliferation markers are TYMS, CCNB1, PTTG1, CEP55, NUF2, CDC20, NDC80, UBE2C, RRM2 and MKI67. **(F)** Violin plot showing the expression of SMC4, TOP2A, FANCB and WEE1 in high/low proliferative LGG patients (n = 530). High or low expression levels are defined with the median. **(G)** The correlation between SMC4, TOP2A, FANCB and WEE1 mRNA levels in LGG patients from TCGA database (n = 530). The size and color of each dot represents correlation coefficient. **(H)** Dot plot and linear regression showing the correlation between the SMC4 mRNA and the WEE1, TOP2A or FANCB mRNA (n = 530). The R square of linear regression model is labeled in the table above the figure. OS, Overall Survival; PFI, Progression-Free Interval; LGG, lower-grade glioma.

Next, we performed univariate and multivariate Cox regression analysis to determine independent genes and/or clinical signatures for the OS or PFI of glioma patients. Multivariate Cox regression was performed with the forward stepwise method (p < 0.05). We identified SMC4, FANCB, WEE1, neoplasm histologic grade and new tumor event after initial treatment as independent markers for the OS, and identified TOP2A, WEE1, mold/dust allergy, neoplasm histologic grade, new tumor event after initial treatment and preoperative corticosteroids as independent markers for PFI (**Figure 2A**). As expected, the histological grade, recurrence after treatment and WEE1 is crucial for both the survival and the PFI of LGG patient. We found that SMC4 and FANCB are independent predictors for survival, and TOP2A, mold/dust allergy and preoperative corticosteroids are independent predictors for the PFI of LGG patients. While there are, limited studies unveiling how the allergy or corticosteroids could affect the PFI of LGG patients, both allergy or corticosteroids indicate the altered immune condition in the tumor microenvironment (such as mast cells), which might affect the prognosis of these patients (27).

Based on the result of multivariate Cox regression model, we calculated the risk score for OS (OS_risk_= 0.882 * SMC4 - 0.759 * FANCB + 0.678 * WEE1 + 1.316 * neoplasm histologic grade + 0.828 * new tumor event after initial treatment) and PFI (PFI_risk_ = -0.132 * TOP2A + 0.533 * WEE1 + 1.667 * mold or dust allergy + 0.795 * neoplasm histologic grade + 2.045 * new tumor event after initial treatment + 0.553 * preoperative corticosteroids), respectively. To assess the efficacy of these risk scores, we ranked all glioma patients with respect to OS_risk_ or PFI_risk_ and defined patients into high or low OS_risk_ or PFI_risk_ according to the median. Kaplan Meier analysis showed both OS_risk_ and PFI_risk_ scores were significantly correlated with the prognosis of the glioma patients (**Figures 2B, C**). Further, we interrogated the prognosis relevance of all four target genes (SMC4, FANCB, WEE1 and TOP2A) enrolled in both risk scores and we observed that all of them are correlated with reduced OS and PFI (**Figure 2D**).

The proliferation potential of LGG patients was assessed with the proliferation signature genes, as previously reported (28), and the top 2 clusters of patients by Hierarchical Clustering were defined as high and low proliferative LGG. Interestingly, SMC4, FANCB, WEE1 and TOP2A were simultaneously elevated in the high proliferation group (**Figures 2E, F**) and all four genes were co-expressed, among which SMC4 showed the highest linear correlation with the rest targets, with all R square > 0.5 (**Figure 2G**).

While all 4 target genes predicted reduced OS and PFI in LGG patients, both FANCB and TOP2A showed a protective role in the risk score model, indicating the controversial effect of FANCB and TOP2A in glioma patients. Besides, the biological and clinical functions of WEE1 have been widely explored. Therefore, we sought to unveil the expression pattern and function of SMC4 in LGG patients.

### The Expression Pattern of SMC4 in LGG Patients

In the Oncomine database, we observed the elevated expression of SMC4 in different cancer types (**Supplementary Figure 2A**) and different glioblastoma databases (**Supplementary Figures 2B–D**). Similarly, we observed the overexpression of SMC4 in 3 different GEO databases (GSE147352, GSE78895 and GSE152071) (**Supplementary Figure 2E**).

**Figure 3:**
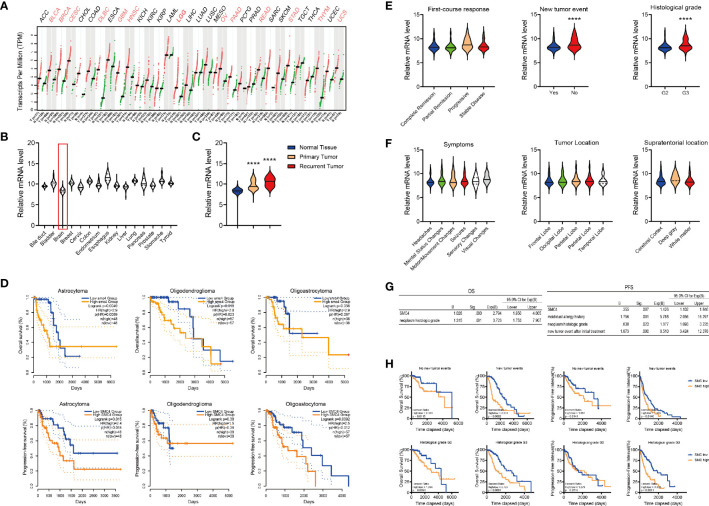
The expression of SMC4 in glioma patients. **(A)** The expression of SMC4 in 32 different cancer types in the GEPIA database. **(B, C)** The expression of SMC4 in normal tissues **(B)** or primary/recurrent tumor tissues **(C)** from the GTEX database. **(D)** Kaplan-Meier analysis comparing the OS and PFI in glioma patients with different histologic origins (n = 97, 75 and 114 for astrocytoma, oligoastrocytoma and oligodendroglioma respectively). Patients were divided into two groups according to SMC4 expression level. **(E)** Violin plot showing the expression of SMC4 in glioma patients with the different first-course responses (the left panel), new tumor events (the middle panel), or histologic staging (the right panel). **(F)** Violin plot showing the expression of SMC4 in glioma patients with different symptoms (the left panel), tumor location (the middle panel), or supratentorial location (the right panel). **(G)** Tables showing the results from the multivariate Cox Regression model for the OS (the left panel) or the PFI (the right panel) with SMC4 and different clinical features in LGG patients. **(H)** Kaplan-Meier analysis comparing the OS and PFI of SMC4 high or low-expressing LGG patients, with or without new tumor events (the top panel) or patients from different histologic staging (the bottom panel). OS, Overall Survival; PFI, Progression-Free Interval; LGG, lower-grade glioma. ****p < 0.0001.

In the GEPIA database, SMC4 is dysregulated in 13 out of 32 types of malignant tumor tissues (**Figure 3A**). As shown in the GTEX database, SMC4 expression in the brain is among the lowest tissues in humans (**Figure 3B**) and is significantly elevated in primary or recurrent tumors (**Figure 3C**). There are three major histological subtypes of glioma (astrocytoma, oligoastrocytoma and oligodendroglioma) and SMC4 overexpression correlated with the reduced OS in astrocytoma and oligodendroglioma (**Figure 3D**, the top panel) and correlated with reduced PFI in astrocytoma and oligo-astrocytoma (**Figure 3D**, the bottom panel).

To decipher the correlation between SMC4 and different clinical phenotypes, we examined the expression of SMC4 mRNA in glioma patients with different first-course responses, new tumor events, histological grades, first symptoms, tumor location and supratentorial locations (**Figures 3E, F**), etc. And results showed SMC4 was only overexpressed in patients with new tumor events and patients with advanced histologic grade (**Figure 3E**).

Then we performed Multivariate Cox Regression with SMC4 and different clinical factors. The result showed SMC4 and neoplasm histologic grade could simultaneously affect the OS of glioma patients, while SMC4 together with mold/dust allergy history, neoplasm histologic grade and new tumor event after initial treatment could independently affect the PFI of the glioma patients (**Figure 3G**). Further, we performed Kaplan-Meier analysis in LGG patients stratifying the new tumor events or histologic grade respectively. Results showed SMC4 could effectively predict the OS and PFI in glioma patients independent of new tumor event(s) (**Figure 3H**, the top panel) or histologic grade (G2 or G3) (**Figure 3H**, the bottom panel).

### SMC4 Expression in Patients With Different Molecular Subtypes

**Figure 4:**
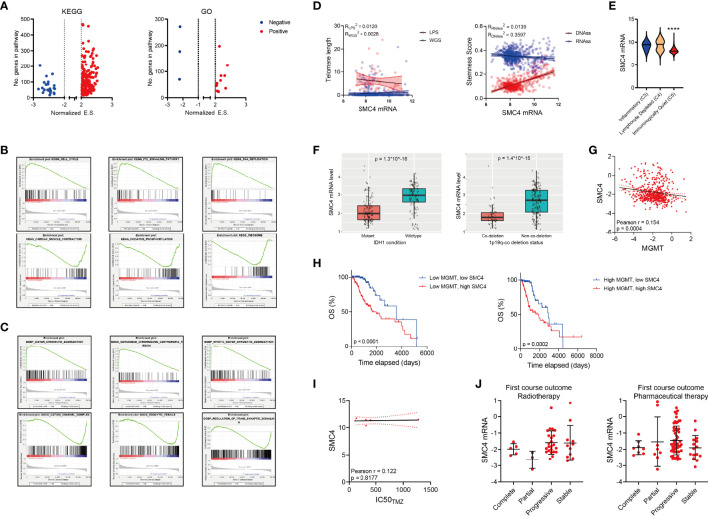
SMC4 expression in patients with different molecular subtypes **(A)** Volcano plot showing the GSEA result comparing the enrichment score (E.S.) of different KEGG (the left panel) or GO (the right panel) pathways in glioma patients with high *vs.* low SMC4 levels. Up-regulated pathways are labeled in red and down-regulated pathways labeled in blue. **(B, C)** GSEA results showing the top 3 activated (the top panel) or suppressed pathways (the bottom panel) in KEGG **(B)** or GO **(C)** pathways. **(D)** Dot plot showing the correlation between SMC4 mRNA level and telomere length calculated with LPS or WGS methods (the left panel) or stemness score (the right panel) calculated based on mRNA or DNA level. The linear regression model with 95% CI were plotted. **(E)** Violin plot showing the expression of SMC4 mRNA in LGG patients with different immune subtypes. **(F)** Bar plot showing the expression of SMC4 mRNA in LGG patients with IDH mutation (the left panel) or 1p19q co-deletion (the right panel). **(G)** Dot plot showing the correlation between SMC4 mRNA and MGMT mRNA in LGG patients. Linear regression and 95% CI are shown in black. **(H)** Kaplan-Meier analysis comparing the survival of high *vs.* low SMC4 in low-MGMT expressing (the left panel) or high MGMT-expressing LGG patients (the right panel). **(I)** Dot plot showing the correlation between the IC50 of TMZ and SMC4 expression level in different LGG cell lines. Linear regression and 95% CI are shown in black. **(J)** Dot plot showing the expression level of SMC4 mRNA in LGG patients undergoing chemotherapy (the right panel) or radiotherapy (the left panel) with different outcomes. GO, Gene Ontology; LGG, lower-grade glioma. ****p < 0.0001.

We performed gene set enrichment analysis (GSEA) to determine the condition of KEGG pathways and GO terms in LGG patients with high or low SMC4 levels. Results showed that majority of the KEGG pathways and GO terms were activated in patients with high SMC4 (**Figure 4A**). The top three activated pathways were cell cycle, p53 signaling and DNA replication in KEGG pathway and sister chromatid segregation, condensed chromosome centromeric region and mitotic sister chromatid segregation in GO terms (**Figures 4B, C**), indicating that SMC4 might participate in the DNA replication and mitosis progression. And the top three suppressed pathways were cardiac muscle contraction, oxidative phosphorylation and ribosome in KEGG pathway and cation channel complex, exocytic vesicle and regulation of trans synaptic signaling in GO terms (**Figures 4B, C**), which indicates the canonical biological function of cells were impaired in SMC4 high expressing LGG tissues.

Cancer cells typically feature abrupt activation of telomerase, upregulated stemness enhanced chemo-resistance to achieve consistent proliferation and avoid senescence or apoptosis (29–31). To determine whether SMC4 could affect these malignant phenotypes, we examined the correlation between SMC4 and the telomere length and stemness scores. While no significant correlation could be observed between SMC4 mRNA and telomere length or mRNA-based stemness score, SMC4 was highly correlated with the DNA-based stemness score (**Figure 4D**). Three major immune conditions were observed in glioma patients and the expression of SMC4 was significantly suppressed in the immunologically quiet patients (**Figure 4E**).

Further, we observed a significant decrease in IDH mutant LGG patients (**Figure 4F**, the left panel) and 1p19q co-deletion LGG patients (**Figure 4F**, the right panel). Yet SMC4 mRNA expression is not significantly correlated with MGMT mRNA (**Figure 4G**) and SMC4 is significantly correlated with the OS of MGMT low-expression and MGMT high-expression LGG patients (**Figure 4H**). Besides, we couldn’t observe any correlation between SMC4 expression and temozolomide (TMZ) sensitivity in LGG patients (**Figure 4I**) and the SMC4 expression is not altered with the prognosis of LGG patients who underwent radiotherapy or pharmaceutical therapy (**Figure 4J**).

### The SMC4 Promotes Proliferation by Repairing Replication Damage in Glioma Cells

**Figure 5:**
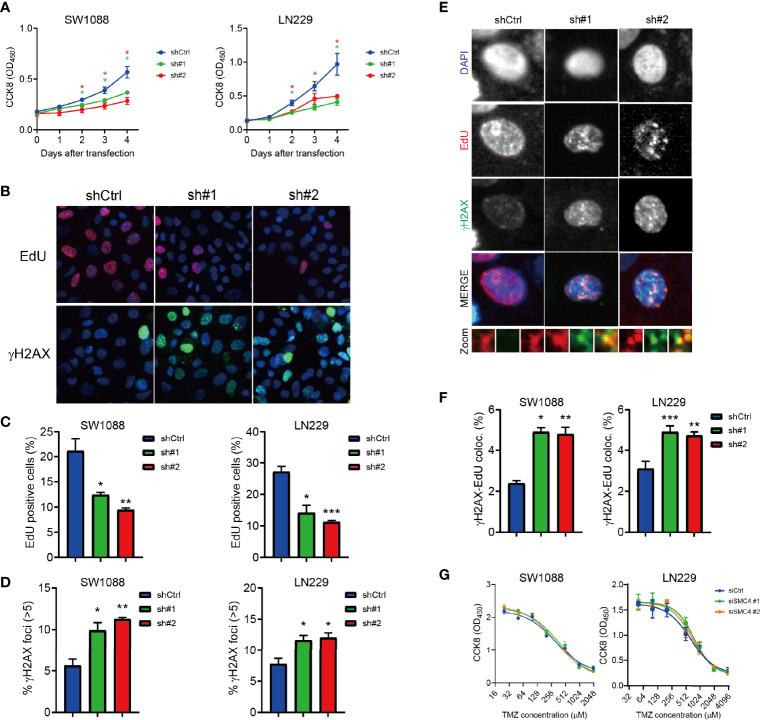
**(A)** Proliferation assay showing reduced proliferation of SMC4 depleted SW1088 and LN229 cells with Cell Counting Kit 8 (CCK8). OD_450_ was measured to quantify cell number in each group. Results from 3 different experiments. **(B–D)** Typical figures **(B)** and quantification **(C, D)** of the EdU staining (**B**, the top panel and **C**) and γH2AX staining (**B**, the bottom panel and **D**) in the control or SMC4 depleted SW1088 and LN229 cells. EdU (red) and γH2AX (green) were detected with immunofluorescence. Result from 3 different experiments. **(E, F)** Typical figures **(E)** and quantification **(F)** of EdU- γH2AX colocalization in the control or SMC4 depleted SW1088 or LN229 cells. EdU (red) and γH2AX (green) were detected with immunofluorescence. Results from 3 different experiments. **(G)** Dose-response analysis comparing the sensitivity of SMC4-depleted SW1088 and LN229 cells to temozolomide (TMZ) after 3 days with CCK8. OD_450_ was measured to quantify cell number in each group. Results from 3 different experiments. Data were represented as mean ± SEM. *p < 0.05; **p < 0.01; ***p < 0.001. The data were analyzed using Student’s t-test.

We established two stable SMC4-depleted cell lines (SW1088 and LN229) with shRNA (**Supplementary Figures 4A, B**). SW1088 and LN229 showed significantly reduced proliferation after SMC4 depletion in the CCK8 assay (**Figure 5A**). We performed EdU staining to reflect the efficacy of DNA replication in cells and γH2AX staining to reflect DNA damage sites. SMC4 depleted SW1088 and LN229 cells exhibited dramatically reduced DNA replication (**Figure 5B**, the top panel and **Figure 5C**). Consistently, we observed increased DNA damage foci in the SMC4 depleted cells (**Figure 5B**, the bottom panel and **Figure 5D**).

Further, we use the colocalization of EdU and γH2AX to reflect the DNA damage in cells and we observed an increased EdU-γH2AX colocalization in the SMC4 depleted cell lines (**Figures 5E, F**). However, we couldn’t observe an increased sensitivity to temozolomide (TMZ) in SMC4 depleted SW1088 cells or LN229 cells (**Figure 5G**) Together, we showed SMC4 could facilitate the repair of DNA replication damage in glioma cells.

### The Transcriptional Modulation of SMC4

**Figure 6:**
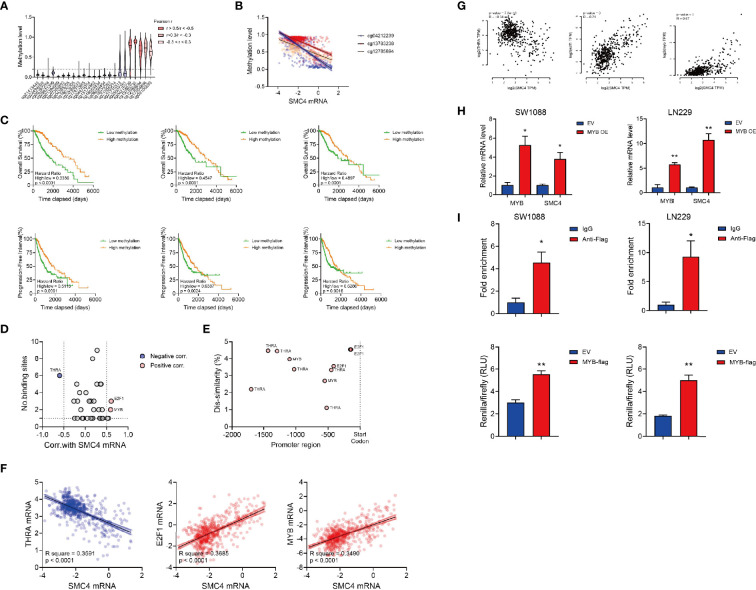
The transcriptional modulation of SMC4. **(A)** Violin plot showing the relative methylation status of SMC4 DNA in glioma patients. **(B)** Dot plot showing the significant correlation between the methylation status and mRNA level of SMC4. cg12785694, cg13783238 and cg04212239 are shown in orange, red or blue. **(C)** Kaplan-Meier analysis comparing the OS and PFI in patients with different SMC4 methylation statuses. Methylation level of cg04212239 (the left panel), cg13783238 (the middle panel) or cg12785694 (the right panel) are analyzed. **(D)** Dot plot showing predicted binding sites on SMC4 promoter region from PROMO database and the correlation between predicted targets and SMC4 level in glioma patients in TCGA database. Pearson correlation -0.5< r <0.5 are labeled in grey; negative correlations (r < -0.5) are labeled in blue and positive correlations (r > 0.5) are labeled in red. **(E)** Dot plot showing the binding site of different targets on SMC4 promoter and its dis-similarity. **(F, G)** Dot plot showing the correlation between SMC4 level and the expression of THRA, E2F1 and MYB in TCGA database **(F)** or GEPIA database **(G)**. **(H)** Quantification of SMC4 and MYB mRNA level in MYB overexpressed SW1088 and LN229 cells with qPCR. GAPDH is used as an internal control for the analysis. Results from 3 different experiments. **(I)** The top panel: quantification of fold enrichment at the promoter region of SMC4 with ChIP-qPCR. The bottom panel: quantification of SMC4 transcription (-800bp~-1000bp) activity with dual-luciferase assay in SW1088 and LN229 cells. Results from 3 different experiments. Data were represented as mean ± SEM. *p<0.05; **p<0.01. The data were analyzed using Student’s t-test.

The genomic methylation level of SMC4 was collected from the TCGA LGG database and SMC4 showed a high methylation level at 5 different domains (**Figure 6A**). We observed 2 intragenic methylation domains (cg12785694 and cg13783238) and 1 promoter region methylation (cg04212239) out of 5 highly methylated domains were negatively correlated with the SMC4 mRNA level (**Figures 6A, B**). And the methylation levels of these 3 domains were positively correlated with the OS and PFI of the glioma patients (**Figure 6C**). While it seemed intriguing that the methylated intragenic domains were negatively correlated with the expression level of SMC4, we noticed that the intragenic methylation could also prevent the initiation of DNA transcription, which might to some extent explain our findings (32). Together, these findings indicate that the transcription of SMC4 is highly dependent on the de-methylation status of these domains. Besides, we observed that the copy number variation of SMC4, either deletion or amplification, could significantly correlated with reduced OS and PFI of LGG patients (**Supplementary Figure 3A**). Yet the mutation of SMC4 couldn’t affect OS and PFI of LGG patients (**Supplementary Figure 3B**), probably due to the limited sample size.

Then we tried to predict the transcription factors responsible for the transcription of SMC4. We predicted the binding dis-similarity of all the known human transcription factors at the SMC4 promoter region (-2000bp) in the PROMO database. The dissimilarity threshold was set to 5% and the correlation between transcription factors and SMC4 was calculated with Pearson correlation. We filtered three transcription factors (THRA, E2F1 and MYB) with more than one binding site on SMC4 promoter were correlated with SMC4 level in TCGA LGG database (Pearson R > 0.4) and their binding sites with the corresponding dis-similarity were plotted (**Figures 6D–F**). Then we validated our findings in the GEPIA database and 3 different GEO databases. In GEPIA, E2F1 and MYB could positively correlate with SMC4 (Pearson R > 0.4) (**Figure 6G**), while in the GEO database, MYB correlates with SMC4 in GSE78895, GSE152071 and GSE147352 (**Supplementary Figure 3C**); E2F1 correlates with SMC4 in GSE147352 (**Supplementary Figure 3D**) and THRA correlates with SMC4 in GSE152071 and GSE147352 (**Supplementary Figure 3E**).

To validate our prediction, we established two Flag-tagged, MYB overexpression glioma cell lines (SW1088 and LN229) and performed Chromatin immunoprecipitation (**Supplementary Figures 4C, D**). We observed the elevated SMC4 in the MYB overexpressed glioma cells (**Figure 6H**). Also, the ChIP assay showed the binding of MYB to the SMC4 promoter at the -976bp~ -837bp region (**Figure 6I**, the top panel). Further, we validated our data with dual-luciferase assay with the -1000bp~-800bp of the SMC4 promoter (**Figure 6I**, the bottom panel).

## Discussion

Lower-grade glioma (LGG) is generally considered to derive from the supporting glial cells of the central nervous system (33). Although glioma is typically considered a chronic disease, the prognosis of LGG has not improved dramatically due to its invasive and infiltrative potential (34–36). Here in this study, we started by demonstrating the expression pattern of LGG patients in a DNA damage repair (DDR) perspective. While LGG is typically considered heterogenous, we noticed a cluster of DDR genes with homogenous expression patterns and defined these genes as the signature for DDR. And it indicates that LGG patients are highly conserved in the expression pattern of DNA damage repair-related genes.

Interestingly, we noticed that the prognosis of LGG patients with higher overall DDR levels is worse than its counterpart. To understand this, we selected independent markers for the prognosis of LGG patients with the Multivariate Cox Regression model and established the corresponding risk score model respectively. Following Kaplan-Meier analysis shows the risk scores for OS and PFI could effectively predict the prognosis of the LGG patients. With bioinformatic analysis, we identified several potential markers for the prognosis of LGG patients, among which SMC4 is identified as a potential target in LGG.

While the work by Jiang et al. has unveiled SMC4 functions to promote glioma proliferation *via* TGF-β pathways, the role of DNA damage repair-related function of SMC4 is not discussed (24). SMC4 is overexpressed in multiple tumor cells, including LGG, hepatocellular carcinoma and breast adenocarcinoma, etc. SMC4 predicts unfavorable outcomes in LGG patients independent of histological subtypes. Although SMC4 is not differentially expressed concerning the symptoms, location and first-course response of the glioma patients, SMC4 is overexpressed in patients with recurrence and advanced histologic staging. Also, SMC4 correlates with reducing OS and PFI in LGG patients despite the new tumor events status or histologic staging. Then we noticed the SMC4 downregulation in IDH mutated and 1p19q co-deleted LGG patients, although the underlying mechanism in between remains to be explored. Besides, SMC4 correlates with the survival of LGG patients, despite MGMT level, which indicates MGMT and SMC4 might function independently in LGG patients.

Multiple KEGG pathways and GO terms are activated in LGG patients with high SMC4 levels, indicating the loss of normal functions and the gain of enhanced proliferation. The methylation status of the SMC4 gene is negatively correlated with the SMC4 mRNA level, which predicts a favorable prognosis of the LGG patients. While it seems intriguing that 2 intragenic domain methylation negatively correlated with SMC4 level, this observation might due to the prevention of spurious transcription initiation by intragenic DNA methylation (32). The binding of transcription factors at the SMC4 promoter region is predicted and MYB and E2F1 are identified as potential transcription factors for SMC4 in glioma patients.

In this study, we illustrated the DDR landscape and identified SMC4 as a potential marker for the prognosis of LGG patients. We demonstrated the expression pattern and the clinical relevance of SMC4 and unveiled the pathway alteration in SMC4 dysregulated LGG patients. Lastly, we explored the methylation status of the SMC4 gene and predicted the potential transcription factors for SMC4 expression. And the limitation of the study is that this is a computational study and further validation on *in vitro* models and LGG patients is needed. While it seems a long way to find a promising approach for LGG patients, we believe SMC4 could be a target for the treatment of glioma patients.

## Data Availability Statement

The original contributions presented in the study are included in the article/**Supplementary Material**. Further inquiries can be directed to the corresponding author.

## Author Contributions

YW and ZW designed the paper. YW and ZW are responsible for the conceptualization of the paper. YW performed the formal analysis. YW and ZW wrote and corrected the manuscript. All authors contributed to the article and approved the submitted version.

## Conflict of Interest

The authors declare that the research was conducted in the absence of any commercial or financial relationships that could be construed as a potential conflict of interest.

## Publisher’s Note

All claims expressed in this article are solely those of the authors and do not necessarily represent those of their affiliated organizations, or those of the publisher, the editors and the reviewers. Any product that may be evaluated in this article, or claim that may be made by its manufacturer, is not guaranteed or endorsed by the publisher.

## References

[1] Yan H Parsons DW Jin G McLendon R Rasheed BA Yuan W. IDH1 and IDH2 Mutations in Gliomas. N Engl J Med (2009) 360:765–73. doi: 10.1056/NEJMoa0808710 PMC2820383 19228619

[2] Youssef G Miller JJ. Lower Grade Gliomas. Curr Neurol Neurosci Rep (2020) 20:21. doi: 10.1007/s11910-020-01040-8 32444979 PMC7244462

[3] Ostrom QT Gittleman H Truitt G Boscia A Kruchko C Barnholtz-Sloan JS. CBTRUS Statistical Report: Primary Brain and Other Central Nervous System Tumors Diagnosed in the United States in 2011-2015. Neuro Oncol (2018) 20:iv1–iv86. doi: 10.1093/neuonc/noy131 30445539 PMC6129949

[4] Paolillo M Boselli C Schinelli S. Glioblastoma Under Siege: An Overview of Current Therapeutic Strategies. Brain Sci (2018) 8(1):15. doi: 10.3390/brainsci8010015 PMC5789346 29337870

[5] Stupp R Mason WP van den Bent MJ Weller M Fisher B Taphoorn MJ. Radiotherapy Plus Concomitant and Adjuvant Temozolomide for Glioblastoma. N Engl J Med (2005) 352:987–96. doi: 10.1056/NEJMoa043330 15758009

[6] Di Carlo DT Cagnazzo F Benedetto N Morganti R Perrini P. Multiple High-Grade Gliomas: Epidemiology, Management, and Outcome. A Systematic Review and Meta-Analysis. Neurosurg Rev (2019) 42:263–75. doi: 10.1007/s10143-017-0928-7 29138949

[7] McNeely T Leone M Yanai H Beerman I. DNA Damage in Aging, the Stem Cell Perspective. Hum Genet (2020) 139:309–31. doi: 10.1007/s00439-019-02047-z PMC6980431 31324975

[8] Yeh CD Richardson CD Corn JE. Advances in Genome Editing Through Control of DNA Repair Pathways. Nat Cell Biol (2019) 21:1468–78. doi: 10.1038/s41556-019-0425-z 31792376

[9] Roos WP Thomas AD Kaina B. DNA Damage and the Balance Between Survival and Death in Cancer Biology. Nat Rev Cancer (2016) 16:20–33. doi: 10.1038/nrc.2015.2 26678314

[10] Li Z Pearlman AH Hsieh P. DNA Mismatch Repair and the DNA Damage Response. DNA Repair (Amst) (2016) 38:94–101. doi: 10.1016/j.dnarep.2015.11.019 26704428 PMC4740233

[11] Jackson SP Bartek J. The DNA-Damage Response in Human Biology and Disease. Nature (2009) 461:1071–8. doi: 10.1038/nature08467 PMC2906700 19847258

[12] Cilona M Locatello LG Novelli L Gallo O. The Mismatch Repair System (MMR) in Head and Neck Carcinogenesis and Its Role in Modulating the Response to Immunotherapy: A Critical Review. Cancers (Basel) (2020) 12(10):3006. doi: 10.3390/cancers12103006 PMC7602801 33081243

[13] McCord M Steffens A Javier R Kam KL McCortney K Horbinski C. The Efficacy of DNA Mismatch Repair Enzyme Immunohistochemistry as a Screening Test for Hypermutated Gliomas. Acta Neuropathol Commun (2020) 8:15. doi: 10.1186/s40478-020-0892-2 32051040 PMC7017562

[14] Pecina-Slaus N Kafka A Salamon I Bukovac A. Mismatch Repair Pathway, Genome Stability and Cancer. Front Mol Biosci (2020) 7:122. doi: 10.3389/fmolb.2020.00122 32671096 PMC7332687

[15] Brown JS O’Carrigan B Jackson SP Yap TA. Targeting DNA Repair in Cancer: Beyond PARP Inhibitors. Cancer Discov (2017) 7:20–37. doi: 10.1158/2159-8290.CD-16-0860 28003236 PMC5300099

[16] Lord CJ Ashworth A. The DNA Damage Response and Cancer Therapy. Nature (2012) 481:287–94. doi: 10.1038/nature10760 22258607

[17] Pandey R Abel S Boucher M Wall RJ Zeeshan M Rea E. Plasmodium Condensin Core Subunits SMC2/SMC4 Mediate Atypical Mitosis and Are Essential for Parasite Proliferation and Transmission. Cell Rep (2020) 30:1883–97.e6. doi: 10.1016/j.celrep.2020.01.033 32049018 PMC7016506

[18] Chen Y Huang F Deng L Yuan X Tao Q Wang T. HIF-1-miR-219-SMC4 Regulatory Pathway Promoting Proliferation and Migration of HCC Under Hypoxic Condition. BioMed Res Int (2019) 2019:8983704. doi: 10.1155/2019/8983704 31828143 PMC6885181

[19] Jinushi T Shibayama Y Kinoshita I Oizumi S Jinushi M Aota T. Low Expression Levels of microRNA-124-5p Correlated With Poor Prognosis in Colorectal Cancer *via* Targeting of SMC4. Cancer Med (2014) 3:1544–52. doi: 10.1002/cam4.309 PMC4298381 25081869

[20] Zhao SG Evans JR Kothari V Sun G Larm A Mondine V. The Landscape of Prognostic Outlier Genes in High-Risk Prostate Cancer. Clin Cancer Res (2016) 22:1777–86. doi: 10.1158/1078-0432.CCR-15-1250 26631616

[21] Zhou B Chen H Wei D Kuang Y Zhao X Li G. A Novel miR-219-SMC4-JAK2/Stat3 Regulatory Pathway in Human Hepatocellular Carcinoma. J Exp Clin Cancer Res (2014) 33:55. doi: 10.1186/1756-9966-33-55 24980149 PMC4096530

[22] Barthel FP Wei W Tang M Martinez-Ledesma E Hu X Amin SB. Systematic Analysis of Telomere Length and Somatic Alterations in 31 Cancer Types. Nat Genet (2017) 49:349–57. doi: 10.1038/ng.3781 PMC5571729 28135248

[23] Zhao Z He K Zhang Y Hua X Feng M Zhao Z. XRCC2 Repairs Mitochondrial DNA Damage and Fuels Malignant Behavior in Hepatocellular Carcinoma. Cancer Lett (2021) 512:1–14. doi: 10.1016/j.canlet.2021.04.026 33964350

[24] Jiang L Zhou J Zhong D Zhou Y Zhang W Wu W. Overexpression of SMC4 Activates TGFbeta/Smad Signaling and Promotes Aggressive Phenotype in Glioma Cells. Oncogenesis (2017) 6:e301. doi: 10.1038/oncsis.2017.8 28287612 PMC5533949

[25] Li Y Jin K van Pelt GW van Dam H Yu X Mesker WE. C-Myb Enhances Breast Cancer Invasion and Metastasis Through the Wnt/beta-Catenin/Axin2 Pathway. Cancer Res (2016) 76:3364–75. doi: 10.1158/0008-5472.CAN-15-2302 27197202

[26] Pearl LH Schierz AC Ward SE Al-Lazikani B Pearl FM. Therapeutic Opportunities Within the DNA Damage Response. Nat Rev Cancer (2015) 15:166–80. doi: 10.1038/nrc3891 25709118

[27] Komi DEA Redegeld FA. Role of Mast Cells in Shaping the Tumor Microenvironment. Clin Rev Allergy Immunol (2020) 58:313–25. doi: 10.1007/s12016-019-08753-w PMC7244463 31256327

[28] Thienpont B Steinbacher J Zhao H D’Anna F Kuchnio A Ploumakis A. Tumour Hypoxia Causes DNA Hypermethylation by Reducing TET Activity. Nature (2016) 537:63–8. doi: 10.1038/nature19081 PMC5133388 27533040

[29] Akincilar SC Unal B Tergaonkar V. Reactivation of Telomerase in Cancer. Cell Mol Life Sci (2016) 73:1659–70. doi: 10.1007/s00018-016-2146-9 PMC4805692 26846696

[30] Cooper J Giancotti FG. Integrin Signaling in Cancer: Mechanotransduction, Stemness, Epithelial Plasticity, and Therapeutic Resistance. Cancer Cell (2019) 35:347–67. doi: 10.1016/j.ccell.2019.01.007 PMC6684107 30889378

[31] Topalian SL Drake CG Pardoll DM. Immune Checkpoint Blockade: A Common Denominator Approach to Cancer Therapy. Cancer Cell (2015) 27:450–61. doi: 10.1016/j.ccell.2015.03.001 PMC4400238 25858804

[32] Neri F Rapelli S Krepelova A Incarnato D Parlato C Basile G. Intragenic DNA Methylation Prevents Spurious Transcription Initiation. Nature (2017) 543:72–7. doi: 10.1038/nature21373 28225755

[33] Delgado-Lopez PD Corrales-Garcia EM Martino J Lastra-Aras E Duenas-Polo MT. Diffuse Low-Grade Glioma: A Review on the New Molecular Classification, Natural History and Current Management Strategies. Clin Transl Oncol (2017) 19:931–44. doi: 10.1007/s12094-017-1631-4 28255650

[34] Duffau H Taillandier L. New Concepts in the Management of Diffuse Low-Grade Glioma: Proposal of a Multistage and Individualized Therapeutic Approach. Neuro Oncol (2015) 17:332–42. doi: 10.1093/neuonc/nou153 PMC4483091 25087230

[35] Sano K Toda M Sasaki H Kitamura Y Mikami S Hirato J. Infratentorial Low-Grade Oligoastrocytoma With Aggressive Clinical Behavior in an Adult: A Case Report With Genetic Characterization. Brain Tumor Pathol (2013) 30:99–103. doi: 10.1007/s10014-012-0111-3 22752622

[36] Wessels PH Weber WE Raven G Ramaekers FC Hopman AH Twijnstra A. Supratentorial Grade II Astrocytoma: Biological Features and Clinical Course. Lancet Neurol (2003) 2:395–403. doi: 10.1016/S1474-4422(03)00434-4 12849117

