# Supplementary material for: The Clinical Significance and Transcription Regulation of a DNA Damage Repair Gene, SMC4, in Low-Grade Glioma via Integrated Bioinformatic Analysis
Source: Front Oncol. 2021 Nov 11;11:761693. doi: 10.3389/fonc.2021.761693 (PMC8636002; doi:10.3389/fonc.2021.761693)
Supplement: Supplementary file 1 [file DataSheet_1.docx]

**Supplementary Material**

**Suppl. Figure 1**


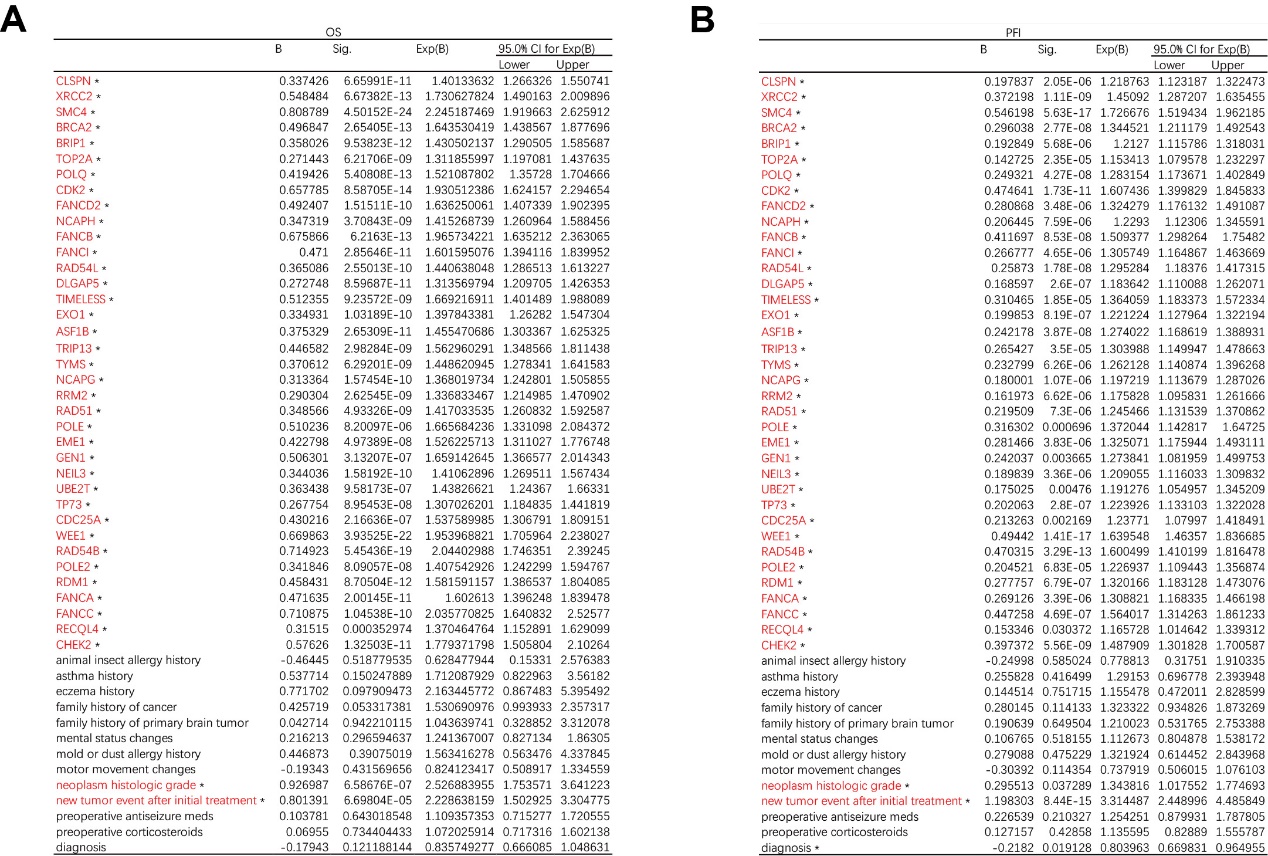


**Univariate Cox Regression model of glioma patients**

(A-B) Results of univariate Cox Regression model for OS (A) or PFI (B) in the LGG patients in TCGA database. Mutual variables were labeled in red.

* significant variables in each analysis.

**Suppl. Figure 2**


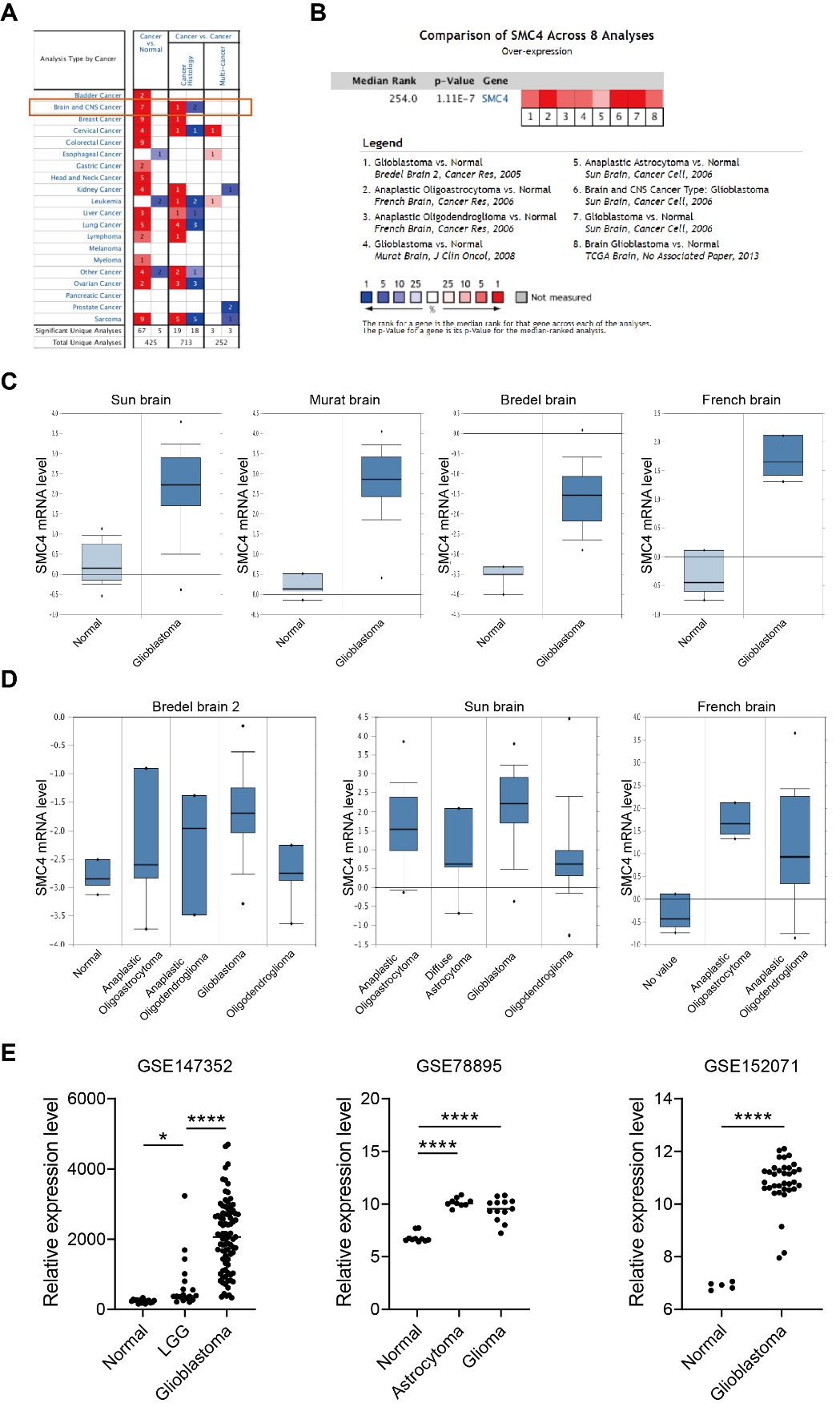


**SMC4 expression level in Oncomine database**

(A) SMC4 expression level in different cancer types.

(B-C) Expression of SMC4 in normal and tumor tissues in different glioblastoma databases.

(D) Expression of SMC4 in different histologic subtypes of glioma tissues.

(E) Expression of SMC4 in different LGG subtypes in GEO database.

**Suppl. Figure 3**


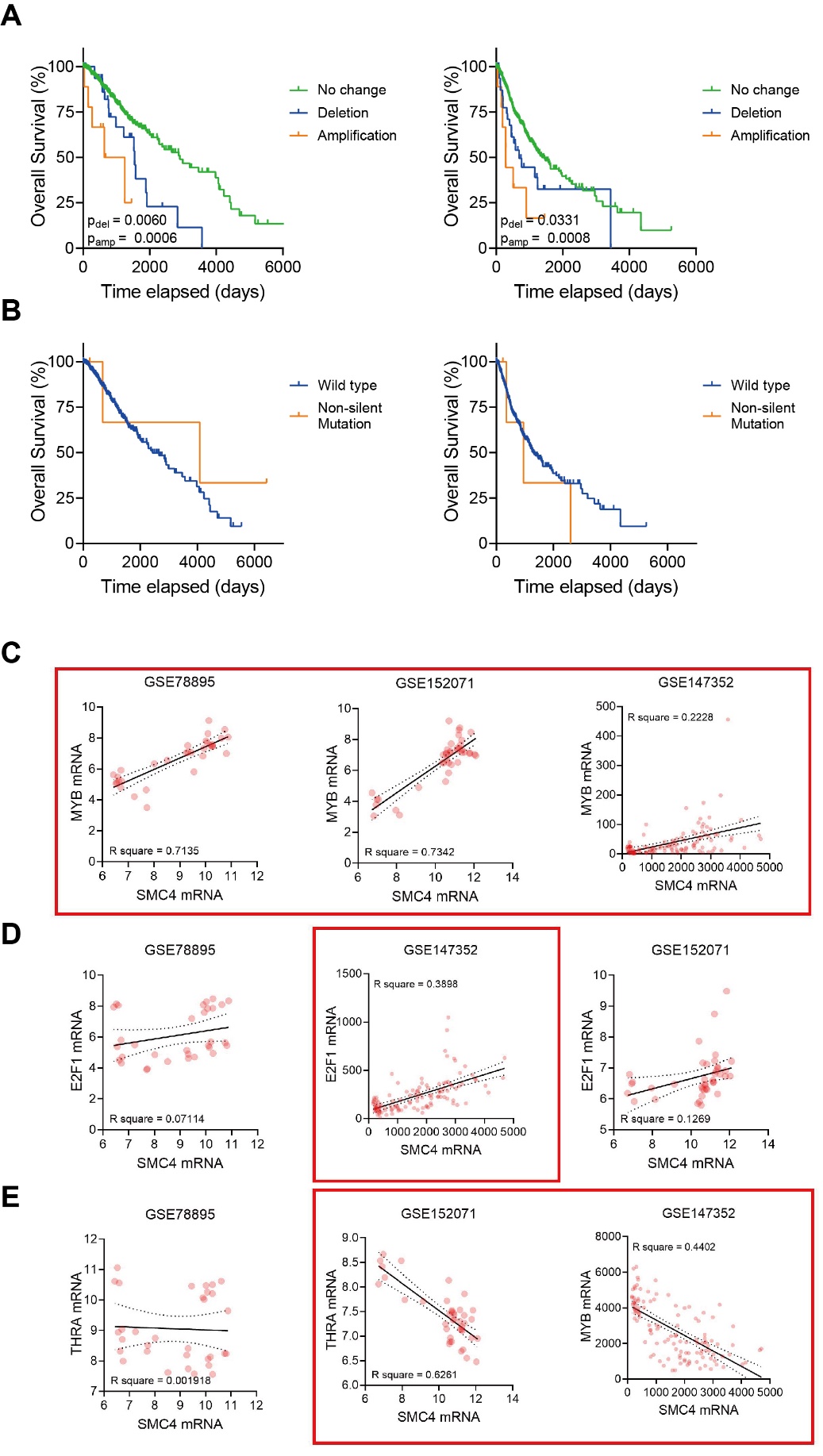


**Methylation and transcription modulation of SMC4**

(A) Kaplan-Meier analysis comparing the overall survival of LGG patients with copy number variation (deletion or amplification) in TCGA LGG patients.

(B) Kaplan-Meier analysis comparing the overall survival of LGG patients with SMC4 mutation in TCGA LGG patients.

(C-E) Correlation between SMC4 mRNA and MYB mRNA (C), E2F1 mRNA (D) and THRA mRNA (E) in 3 GEO databases. Linear regression was performed and groups with Pearson R > 0.4 was labeled in red square.

**Suppl. Figure 4**


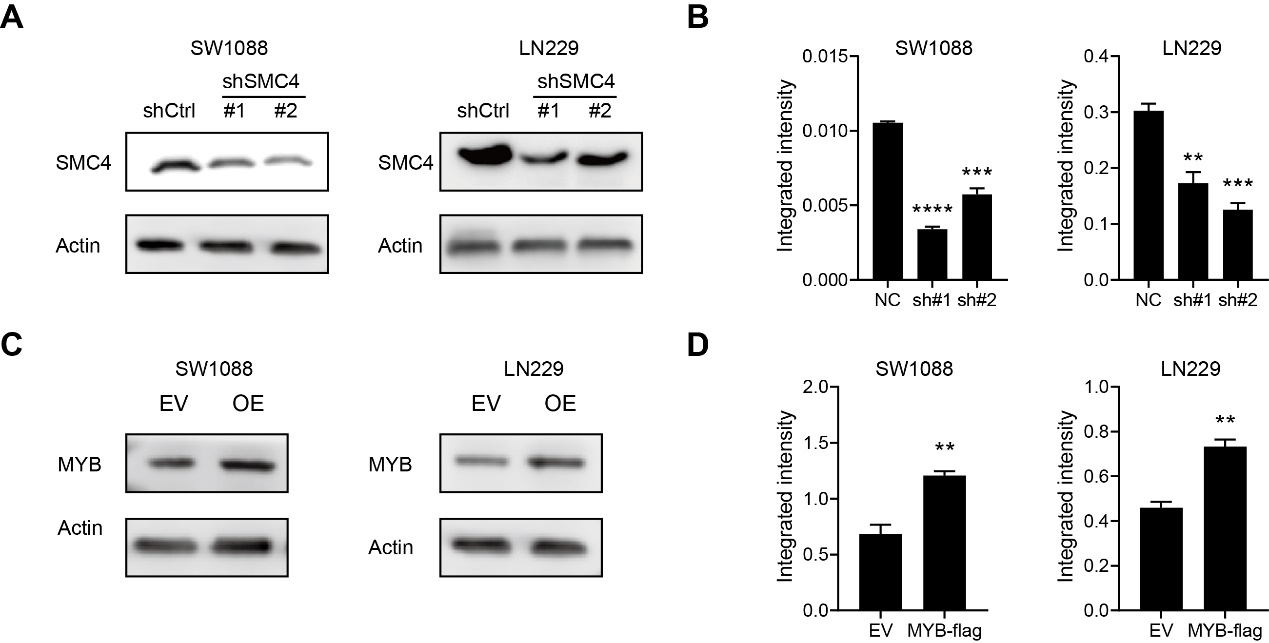


**ShRNA and MYB overexpression**

(A-B) Immunoblots (A) and quantification (B) of SMC4 level in SW1088 and LN229 cells transfected with non-targeting shRNA or shSMC4 #1 or #2.

(C-D) Immunoblots (C) and quantification (D) of MYB level in SW1088 and LN229 cells overexpressing empty vector or MYB-flag.

Results from 3 different experiments. Data were represented as mean ± SEM. **p＜0.01, ***p＜0.001, ****p＜0.0001. The data were analyzed using Student’s t-test.
